# How Will Our Practice Change After the CLEAR Outcomes Trial?

**DOI:** 10.1007/s11883-024-01188-5

**Published:** 2024-01-31

**Authors:** Timothy Abrahams, Adam J. Nelson, Stephen J. Nicholls

**Affiliations:** Victorian Heart Institute, MonashUniversity, 631 Blackburn Road, Clayton, VIC 3168 Australia

**Keywords:** Lipid lowering, Cardiovascular risk, Bempedoic acid, Statin intolerance

## Abstract

**Purpose of Review:**

Bempedoic acid is a novel therapeutic agent that is designed to reduce levels of low-density lipoprotein cholesterol (LDL-C). The purpose of this review is to provide the background for development of bempedoic acid, findings from clinical trials and to discuss clinical implications.

**Recent Findings:**

Bempedoic acid inhibits ATP citrate lyase within the liver and reduces cholesterol synthesis, with the potential to avoid muscle symptoms experienced by patients treated with statins. Early clinical studies demonstrated that administration of bempedoic acid resulted in lowering of LDL-C by 20–30% as monotherapy and by 40–50% when combined with ezetimibe, in addition to lowering of high sensitivity C-reactive protein by 20–30%. The CLEAR Outcomes trial of high cardiovascular risk patients, with elevated LDL-C levels and either unable or unwilling to take statins demonstrated that bempedoic acid reduced the rate of major adverse cardiovascular events. A greater incidence of elevation of hepatic transaminase and creatinine, gout, and cholelithiasis were consistently observed in bempedoic acid–treated patients.

**Summary:**

Bempedoic acid presents an additional therapeutic option to achieve more effective lowering of LDL-C levels and reduction in cardiovascular risk.

## Introduction

Clinical trials have consistently demonstrated that lowering levels of low-density lipoprotein cholesterol (LDL-C) produces reductions in cardiovascular event rates in high risk patients [1–9, 10••]. The results of these trials have transformed approaches to the prevention of cardiovascular disease [11, 12]. However, the finding that many individuals fail to achieve the degree of LDL-C lowering required for effective risk reduction suggests that additional approaches are required. This has led to the development of a range of new therapies for use in clinical practice.

## LDL and Cardiovascular Risk

An abundant body of evidence has established that LDL-C plays a causal role in atherosclerotic disease. Early observations from cohort studies have been supported by the findings of both preclinical investigation and Mendelian randomization to demonstrate that higher LDL-C levels associate with a greater risk of atherosclerotic disease, supporting the need for therapeutic interventions to lower circulating levels of atherogenic lipid parameters [13]. Clinical trials of statin therapy demonstrated that lowering LDL-C levels associated with reductions in cardiovascular event rates in the primary and secondary prevention settings [1–6] and slow progression of coronary atherosclerosis [14–16]. More recent studies that compared the effects of more and less intensive statin therapy resulted in greater reduction in cardiovascular risk, associated with achieving lower LDL-C levels [6]. On the basis of these findings, prevention guidelines have been successively updated to highlight the importance of intensive lipid lowering, with statin therapy as the cornerstone, to achieve more effective prevention of cardiovascular events [11, 12].

The finding that many patients continue to experience cardiovascular events, despite statin treatment, supports the need for additional lipid-lowering agents in clinical practice [17]. Clinical trials have subsequently demonstrated the clinical benefit of adding either ezetimibe [7] or proprotein convertase subtilisin kexin type 9 (PCSK9) inhibitors [8, 9] to statin therapy. This supports the benefits of combination therapy to produce more effective lipid lowering in a greater proportion of patients, resulting in less clinical events. Ongoing investigation has been undertaken to develop novel lipid-lowering agents that can be employed as either monotherapy or in addition to statin therapy.

## Bempedoic Acid

**Fig. 1:**
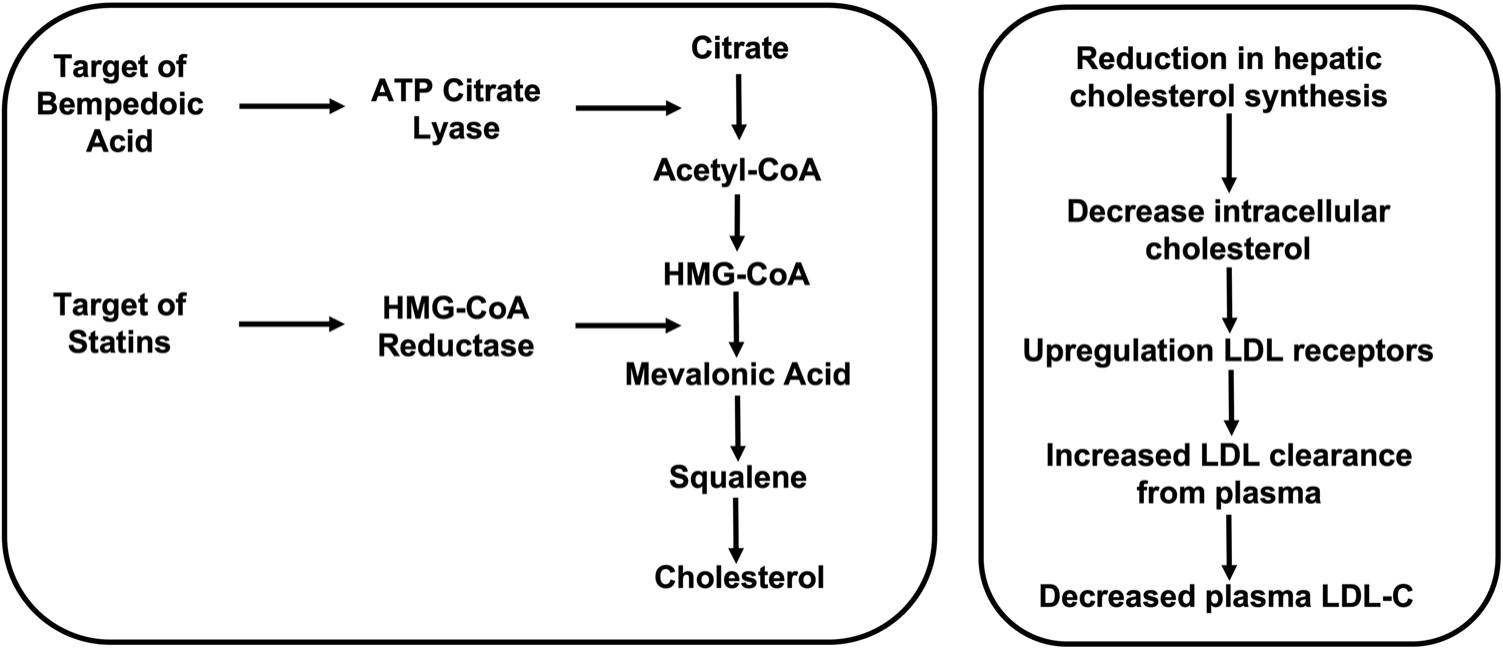
Mechanism of bempedoic acid and statins in lipid lowering. Mechanism of bempedoic acid and statins in lowering levels of low-density lipoprotein cholesterol (LDL-C). Both agents lead to a reduction in hepatic intracellular cholesterol levels, with consequent upregulation of surface LDL receptor expression, greater LDL clearance, and a reduction in plasma LDL-C levels. ATP, adenosine triphosphate; HMG-CoA, hydroxy methyl glutaryl coenzyme A

Bempedoic acid is an oral inhibitor of ATP citrate lyase (ACL), a factor involved in the same cholesterol synthesis pathway within the liver as hydroxy methyl glutaryl coenzyme A (HMGCoA) reductase, the target of statins [18]. Accordingly, inhibition of ACL should similarly result in reductions in hepatic cholesterol synthesis and an increase in hepatocyte surface expression of the LDL receptor, resulting in greater removal of LDL-C from the circulation (Figure 1). Mendelian randomization studies have established that polymorphisms resulting in less ACL associate with a reduction in cardiovascular risk, directly proportional to the degree of lowering of either LDL-C or apolipoprotein (apo) B [19]. This would suggest that ACL inhibition has the potential to be a promising agent for both lowering LDL-C levels and the prevention of cardiovascular disease.

Bempedoic acid is an oral, small molecule inhibitor of ACL. It is ingested in an inactive form and requires activation by the isozyme ACSVL1 which is present in the liver, but not skeletal muscle [18, 20, 21]. In theory, this provides the potential for lipid lowering, without the myalgia that is experienced by many patients treated with a statin. Early clinical studies demonstrated that treatment with bempedoic acid produced reductions in LDL-C by 20–30% as monotherapy [22–29] and by 40–50% [30] when used in combination with ezetimibe, in patients with and without statin associated muscle symptoms. This led to a large phase 3 clinical development program: Cholesterol Lowering via Bempedoic Acid, an ACL-Inhibiting Regimen (CLEAR) to comprehensively evaluate the impact of bempedoic acid on lipid levels and cardiovascular outcomes.

Monotherapy studies of bempedoic acid have been conducted across the spectrum of statin tolerance. The CLEAR Harmony [27] and Wisdom [28] studies evaluated the impact of bempedoic acid in patients treated with either high intensity (51%) or low to moderate intensity (49%) statin therapy. These studies demonstrated placebo-corrected reductions in LDL-C with bempedoic acid by 18.1% and 17.4%, respectively. CLEAR Serenity [29] evaluated the impact of bempedoic acid in patients meeting a traditional definition for statin intolerance, requiring documented failure of two statins, one at the lowest available dose. Placebo-corrected reductions in LDL-C by bempedoic acid by 21.4% were reported. CLEAR Tranquility also involved patients with statin intolerance, but employed a more pragmatic definition for statin intolerance, permitting recruitment of patients who had failed only one statin at any dose. In this study, placebo-corrected reductions in LDL-C of 28.5% were observed with bempedoic acid [26]. Reductions in high sensitivity C-reactive protein (hsCRP) by 18.7–32.5% were also observed in these studies. These early studies demonstrated that treatment with bempedoic acid was associated with a greater incidence of liver transaminase and creatinine elevations, gout and cholelithiasis. The CLEAR Harmony and Wisdom studies reported the incidence of tendon rupture in 10 patients treated with bempedoic acid compared with 0 in the placebo groups.

A combination study of 382 patients with atherosclerotic cardiovascular disease, heterozygous familial hypercholesterolemia, or the presence of multiple cardiovascular risk factors, treated with maximally tolerated statin therapy, compared the effects of treatment with placebo, ezetimibe, and bempedoic acid 180 mg as monotherapy or in combination with ezetimibe for 12 weeks. Placebo-corrected reductions in LDL-C were observed with ezetimibe by 25%, bempedoic acid by 19% and the combination by 38% [30]. Reductions in hsCRP levels by 35.1% were observed with the combination of bempedoic acid and ezetimibe [30]. An additional study of the combination of bempedoic acid, ezetimibe, and atorvastatin 20 mg daily produced reductions in LDL-C by 63.6% and hsCRP by 47.7% [31].

## CLEAR Outcomes

The CLEAR Outcomes study was conducted in 13,970 high cardiovascular risk patients with elevated LDL-C levels and documented statin intolerance. Patients were required to have clinically manifest atherosclerotic cardiovascular disease or be deemed to be at high risk of having a cardiovascular event with a LDL-C at least 100 mg/dL (2.6 mmol/L). All patients were required to have statin intolerance, defined as having experienced an adverse effect that started or increased during statin therapy and resolved or improved after therapy was discontinued. Patients were required to have experienced intolerance to at least two statins or one if they were unwilling to attempt a second statin or they were advised by a physician to not attempt a second statin. All patients and investigators were required to document that they were unable to tolerate medications that have been documented to reduce the risk of heart attack and stroke. Patients treated with very low doses of statins, less than the daily approved doses, were permitted to participate in the study. Patients were treated with bempedoic acid 180 mg or matching placebo daily and followed for at least 24 months and until at least 1620 primary endpoints (cardiovascular death, nonfatal myocardial infarction, nonfatal stroke and coronary revascularization) and 810 key secondary endpoints (cardiovascular death, nonfatal myocardial infarction, and nonfatal stroke) occurred [10••, 32].

Patients were recruited at 1250 sites in 32 countries from December 2016 to August 2019 and were followed for a median duration of 40.6 months, experiencing 1746 primary endpoints and 1238 key secondary endpoints. Baseline demographics included mean age 65 years, 48% female sex, 70% secondary prevention, 45% diabetes, LDL-C 139 mg/dL (3.5 mmol/L), and hsCRP 2.3 mg/L. Low-dose statins were used in 22% of patients. At 6 months, bempedoic acid produced greater lowering of LDL-C (− 21.7 vs − 0.6%) and hsCRP (− 22.2 vs + 2.4%) compared with placebo. Over the course of the study, the LDL-C levels between groups narrowed, as a result of disproportionate cross in of lipid-lowering agents in the placebo group [10••].

For the primary composite endpoint, a hazard ratio in favor of benefit with bempedoic acid was observed (HR 0.87, 95% CI 0.79–0.96, *P* = 0.004) was observed. This translated to an absolute risk reduction of 1.6% and number needed to treat to prevent one event of 63. For the key secondary composite endpoint a similar benefit was observed in favor of benefit with bempedoic acid (HR 0.85, 95% CI 0.76–0.96, *P* = 0.006). For both of these endpoints, event curves began to separate after 9 months, consistent with prior lipid-lowering trials. Similarly, reductions in the incidence of fatal and nonfatal myocardial infarction (HR 0.77, 95% CI 0.66–0.91, *P* = 0.002) and coronary revascularization (HR 0.81, 95% CI 0.72–0.92, *P* = 0.001) were also observed with bempedoic acid. In contrast, no significant reduction in stroke, all cause or cardiovascular mortality was observed [10••].

No difference was observed between the groups with regard to the incidence of adverse events, discontinuation of study drug or muscle events. Patients treated with bempedoic acid experienced a greater incidence of elevation of liver transaminases or creatinine, gout or cholelithiasis. These events were mild and rapidly resolved with discontinuation of study drug. On the basis of the finding from early studies, CLEAR Outcomes prespecified formal adjudication of tendon rupture events and found no difference in its incidence between treatment groups [10••].

## Primary Prevention, Total Events, Diabetes, CTT

Subgroup analysis of the primary composite endpoint revealed no evidence of treatment heterogeneity. However, it did suggest potentially greater cardiovascular benefit with bempedoic acid in patients without clinically manifest atherosclerotic cardiovascular disease at study entry [10••]. These patients were recruited on the basis of having a Reynolds Risk score > 30%, SCORE risk > 7.5% over 10 years, diabetes with age > 65 years in women or > 60 years in men or a coronary calcium score > 400 Hounsfield units. The primary prevention cohort were more likely to be female (59%) and have diabetes (66%) with similar reductions in LDL-C and hsCRP with bempedoic acid. Greater reductions in the primary endpoint (HR 0.70, 95% CI 0.55–0.89, *P* = 0.002), key secondary endpoint (HR 0.64, 95% CI 0.48–0.84, *P* < 0.001) and myocardial infarction (HR 0.61, 95% CI 0.39–0.98) were observed with bempedoic acid. In contrast to the overall findings of the study, the primary prevention cohort demonstrated significant reductions in cardiovascular (HR 0.61, 95% CI 0.41–0.92) and all-cause (HR 0.73, 95% CI 0.54–0.98) mortality with bempedoic acid [33••].

Additional analyses of CLEAR Outcomes have demonstrated that the extent of cardiovascular risk reduction for the degree of LDL-C lowering fit on the Cholesterol Treatment Trialist’s curve for the secondary prevention cohort and was greater for those without clinically manifest disease at baseline (https://www.hcplive.com/view/clear-outcomes-data-at-endo-2023-provides-further-insight-into-effects-of-bempedoic-acid [https://www.hcplive.com/view/clear-outcomes-data-at-endo-2023-provides-further-insight-into-effects-of-bempedoic-acid]). Greater clinical benefit was observed for bempedoic acid on total events analysis and in patients with diabetes at baseline [34, 35]. No adverse effect was observed on measures of glycemic control and there was no greater likelihood of developing new onset diabetes during the study [35]. These findings extend the benefits of bempedoic acid on LDL-C and hsCRP to reductions in the risk of cardiovascular events.

## Implications for Statin Intolerance

CLEAR Outcomes represents the largest clinical trial conducted in patients who were unable or unwilling to take approved doses of statins. Clinical registries of high cardiovascular risk patients demonstrate that at least 50% of patients fail to be treated with intensive statin therapy [36] and that 50% of patients prescribed a statin will cease therapy within the next 18 months [37, 38]. Predictably, reduced adherence to statin therapy will have implications for suboptimal reductions in LDL-C and cardiovascular risk [39]. A major factor contributing to either discontinuation or use of appropriate doses of statin therapy is the incidence of symptoms of intolerance, with an incidence of at least 10% in cohort studies [40]. The true mechanism underlying the experience of statin associated muscle symptoms remains uncertain, with some evidence that the nocebo effect may play an important role [41]. Nevertheless, patients unable to tolerate appropriate doses of statin therapy fail to achieve the degree of lipid lowering required to reduce their cardiovascular risk.

A number of therapeutic options currently exist for the management of these patients. Attempts at using lower doses of statins, at less than daily frequency of administration, has been reported to produce LDL-C lowering by more than 20% in patients [42]. Similarly, use of either ezetimibe or PCSK9 inhibitors [43] has been demonstrated to produce effective LDL-C lowering in patients with statin intolerance and both have been reported to produce reductions in cardiovascular risk in large outcomes trials [7–9]. The finding of CLEAR Outcomes provides definitive evidence that use of bempedoic acid effectively lowers both LDL-C and hsCRP, in addition to reducing cardiovascular risk in patients who are unable or unwilling to take a statin. This provides important evidence of a clinical pathway that could be effectively used for these patients.

## Implications for Combination Therapy

The studies within the CLEAR development program establish the efficacy, safety, and tolerability data to guide use of bempedoic acid in clinical practice. While the utility in patients with statin intolerance is supported by the benefits of the cardiovascular outcomes trial, the potential to incorporate bempedoic acid within combination lipid lowering algorithms may provide a much greater therapeutic opportunity. Cohort studies of high cardiovascular risk patients consistently report a low rate of use of combination therapy, albeit with a trend toward increasing utilization [36, 44]. However, with progressive updates to treatment guidelines, advocating lower LDL-C goals, a greater proportion of patients will require use of combination therapy to reach these targets. This will require treatment of dyslipidemia to become similar to that of hypertension and type 2 diabetes, in which use of combination therapy has become commonplace.

## Implications for Primary Prevention and High Risk

The additional analyses of the CLEAR Outcomes trial provide further information regarding the modifiability of cardiovascular risk. The finding of greater benefit in the primary prevention patients highlights the potential to substantially lower the risk of cardiovascular events in patients who are deemed to be at high risk, yet have not experienced an adverse clinical outcome. This supports similar findings of benefit of statins in high risk primary prevention cohorts and highlights the potential modifiability in these patients [45, 46]. Similarly, the finding of greater absolute benefit from the perspective of total events and in patients with diabetes further identifies the ability to target more intensive therapy to patients at greater risk, which has important health and economic implications.

## Future Directions

The benefits of bempedoic acid further highlight the benefits of lipid lowering in reducing cardiovascular risk. Additional work is required to implement the findings of these studies into clinical practice. In addition, ongoing development programs are evaluating the therapeutic potential of novel LDL-C lowering agents including both oral [47] and new injectable [48] PCSK9 inhibitors, ongoing attempts to determine if cholesteryl ester transfer protein inhibitors reduce cardiovascular risk [49] and the emergence of gene editing interventions [50] that have the potential to produce once in a lifetime approaches to lipid lowering. With an increasing toolbox of therapeutic strategies to lower LDL-C there is a greater potential to achieve effective lipid lowering in the majority of high risk patients. The ongoing challenge to maintain long term adherence with therapy requires development of novel models of care focusing on both patients and their treating health care professionals.

## Conclusions

Bempedoic acid provides an additional approach to lipid lowering with unequivocal data from clinical studies demonstrating its efficacy in improving lipid profiles and reducing cardiovascular risk. Whether administered as monotherapy in patients who are either unwilling or unable to take statins or in combination with other lipid-lowering agents, it provides another option in clinical practice to achieve more effective control of LDL-C and enhance our ability to prevent cardiovascular events.
